# Characterising the biophysical, economic and social impacts of soil carbon sequestration as a greenhouse gas removal technology

**DOI:** 10.1111/gcb.14844

**Published:** 2019-10-26

**Authors:** Alasdair J. Sykes, Michael Macleod, Vera Eory, Robert M. Rees, Florian Payen, Vasilis Myrgiotis, Mathew Williams, Saran Sohi, Jon Hillier, Dominic Moran, David A. C. Manning, Pietro Goglio, Michele Seghetta, Adrian Williams, Jim Harris, Marta Dondini, Jack Walton, Joanna House, Pete Smith

**Affiliations:** ^1^ Scotland's Rural College (SRUC) Edinburgh UK; ^2^ School of Geosciences The University of Edinburgh Edinburgh UK; ^3^ Global Academy of Agriculture and Food Security The University of Edinburgh Midlothian UK; ^4^ School of Natural and Environmental Sciences Newcastle University Newcastle‐upon Tyne UK; ^5^ School of Water, Energy and Environment Cranfield University Bedford UK; ^6^ Institute of Biological & Environmental Sciences University of Aberdeen Aberdeen UK; ^7^ Cabot Institute University of Bristol Bristol UK

**Keywords:** 4 per mille, agriculture, greenhouse gas removal, negative emissions, soil carbon sequestration, soil organic carbon

## Abstract

To limit warming to well below 2°C, most scenario projections rely on greenhouse gas removal technologies (GGRTs); one such GGRT uses soil carbon sequestration (SCS) in agricultural land. In addition to their role in mitigating climate change, SCS practices play a role in delivering agroecosystem resilience, climate change adaptability and food security. Environmental heterogeneity and differences in agricultural practices challenge the practical implementation of SCS, and our analysis addresses the associated knowledge gap. Previous assessments have focused on global potentials, but there is a need among policymakers to operationalise SCS. Here, we assess a range of practices already proposed to deliver SCS, and distil these into a subset of specific measures. We provide a multidisciplinary summary of the barriers and potential incentives towards practical implementation of these measures. First, we identify specific practices with potential for both a positive impact on SCS at farm level and an uptake rate compatible with global impact. These focus on: (a) optimising crop primary productivity (e.g. nutrient optimisation, pH management, irrigation); (b) reducing soil disturbance and managing soil physical properties (e.g. improved rotations, minimum till); (c) minimising deliberate removal of C or lateral transport via erosion processes (e.g. support measures, bare fallow reduction); (d) addition of C produced outside the system (e.g. organic manure amendments, biochar addition); (e) provision of additional C inputs within the cropping system (e.g. agroforestry, cover cropping). We then consider economic and non‐cost barriers and incentives for land managers implementing these measures, along with the potential externalised impacts of implementation. This offers a framework and reference point for holistic assessment of the impacts of SCS. Finally, we summarise and discuss the ability of extant scientific approaches to quantify the technical potential and externalities of SCS measures, and the barriers and incentives to their implementation in global agricultural systems.

## Graphical abstract

To limit warming to well below 2°C, many scenario projections rely on greenhouse gas removal by soil carbon sequestration (SCS) in agricultural land. Here, we provide a multidisciplinary summary of the barriers and potential incentives towards practical implementation SCS. First, we identify specific practices with potential for both a positive impact on SCS and an uptake rate compatible with global impact. Then, we then consider economic and non‐cost barriers and incentives for implementing SCS, and potential externalised impacts of implementation. Finally, we discuss the ability of existing methods to model the potential of SCS in global agricultural systems.

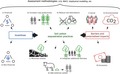

**Abbreviations**
AR: afforestation/reforestation
BAU: business‐as‐usual [scenario]
BECCS: bioenergy with carbon capture and storage
DAC: direct air capture
EW: enhanced weathering
GGR: greenhouse gas removal
GGRT: greenhouse gas removal technology
GHG: greenhouse gas
IAM: integrated assessment model
IPCC: Intergovernmental Panel on Climate Change
LCA: life cycle assessment
MRV: monitoring, reporting and verification
NPK: nitrogen, phosphorus, potassium [fertiliser]
OM: organic matter
SCS: soil carbon sequestration
SDG: sustainable development goals
SOC: soil organic carbon

## 1 INTRODUCTION

Despite concerted international effort to curb greenhouse gas (GHG) emissions, their release to the atmosphere accelerated throughout the first decade of the 21st century (Le Quéré et al., 2012). The adoption of the Paris Agreement represented an international consensus to limit global temperature rise to well below 2°C above pre‐industrial levels and an ambition to limit to 1.5°C (United Nations Framework Convention on Climate Change, 2015). To meet the 2°C target, Fuss et al. (2014) estimated that cumulative emissions from 2015 must be restricted to 1,200 Gt CO_2_. Most integrated assessment models (IAMs) rely on GHG removal technologies (GGRTs) to have a greater than 50% chance of achieving this (Riahi et al., 2017; Rogelj et al., 2018; Smith, Davis, et al., 2016; Smith, Grant, et al., 2016). The GGRT literature is still in relative infancy, but is growing fast and recognition of the need for the wide‐scale deployment of GGRTs is increasing (Fuss et al., 2014, 2018; Minx, Lamb, Callaghan, Bornmann, & Fuss, 2017; Minx et al., 2018; Popp et al., 2017; Rogelj et al., 2018).

Several GGRTs are under consideration; the most prevalent are bioenergy with carbon capture and storage (BECCS), direct air capture (DAC), enhanced weathering (EW), afforestation/reforestation (AR) and soil carbon sequestration (SCS; Fuss et al., 2018; Minx et al., 2018; Popp et al., 2017; Smith, 2016; Smith, Davis, et al., 2016; Smith, Grant, et al., 2016). SCS shows several important advantages over other GGRTs (Smith, 2016); it has negligible land use impacts since it can be practised without changing land use (a drawback of BECCS and AR). Besides GGRTs, land‐based measures such as reduced impact logging can achieve mitigation with negligible land use change (Ellis et al., 2019). SCS implementation costs are estimated to be negative for around 20% of potential, and <US$ 40 t/C‐eq for the remainder, making it highly cost‐effective versus DAC and EW (Smith, 2016). Water and energy use by SCS are negligible or negative, providing an advantage over BECCS, DAC and AR (Smith, 2016). A key limitation of SCS is saturation of sequestration potential, making GGR by SCS a finite and time‐limited quantity, and vulnerable to reversal (Fuss et al., 2014). The global potential of SCS is also challenging to assess, and optimistic assessments are disputed (Schlesinger & Amundson, 2019). While the estimated global potential of SCS is lower than some other GGRTs (Fuss et al., 2018; Minx et al., 2018; Smith, 2016), the efficacy of SCS is greatest in the short to medium term (Goglio et al., 2015; Smith, 2012), meaning SCS may act as an interim measure until the deployment of higher potential GGRTs can be realised.

Conversion of undisturbed land to agriculture typically results in a loss of SOC (Paustian et al., 2016; Six, Conant, Paul, & Paustian, 2002). This human activity has a pedigree of 12 millennia, dating to the agricultural revolution of the early Holocene (Klein Goldewijk, Beusen, Drecht, & Vos, 2011). Thus, a considerable carbon ‘debt’ has been accrued, estimated at 133 Pg C (Sanderman, Hengl, & Fiske, 2017). Within the context of SCS, this debt represents a sequestration opportunity, as agricultural soils may have the capacity to regain historically lost C.

Soil carbon sequestration can play a critical role in delivering improved soil quality and food security (Fuss et al., 2018; Paustian et al., 2016; Smith, 2016), and is therefore a key contributor to sustainable development goals (Chabbi et al., 2017; Keesstra et al., 2016). Additionally, it is integral to the large‐scale ecosystem restoration requirements highlighted by international bodies (IPBES, 2018). This, coupled with the negative to low cost of SCS implementation, makes it a no‐regrets option, and growing recognition of this is reflected in its incorporation into international initiatives such as the 4 per mille (4‰) proposition (Minasny et al., 2017).

Heterogeneity in environmental conditions and agricultural practices challenge the practical implementation of SCS measures (Lal, Negassa, & Lorenz, 2015). This complexity, coupled with the low per‐area abatement potential, means that SCS has received comparatively little attention in the GGRT IAM scenarios literature (Popp et al., 2017; Riahi et al., 2017). While several SCS reviews have been conducted, these have typically been either region‐specific (Luo, Wang, & Sun, 2010; Merante et al., 2017; Vågen, Lal, & Singh, 2005), practice‐specific (Lehmann, Gaunt, & Rondon, 2006; Lorenz & Lal, 2014; McSherry & Ritchie, 2013) or have assessed global potentials without considering explicitly the practices used to deliver SCS (Fuss et al., 2018; Griscom et al., 2017; Smith, 2016). Some broader reviews have been conducted (e.g. Stockmann et al., 2013), though the pace at which scientific knowledge is advancing in this field (Minx et al., 2017) merits a continuation and enhancement of this process. Since soil forms an integral part of the vast majority of agricultural systems, SCS measures must necessarily impact the agroecosystem as a whole, and this impact may directly affect the wider social and economic systems to which the agroecosystem is linked. The biophysical complexity of SCS is thus compounded by inextricable socio‐economic complexities. Consequently, in order to facilitate GGR via SCS, measures must be implemented which inherently have:

1. uncertainty relating to technical abatement rate and potential;
2. uncertainty relating to costs; and
3. the potential to induce a range of impacts on the agroecosystem in question.
4. As a result of 3, the potential to induce further impacts on the wider social and economic systems which are linked, directly or indirectly, to the agroecosystem in question.

For many measures, the extant literature is in a position to provide answers to each of these elements. What is lacking is a framework which brings this literature together in a coordinated and comparable way. This paper seeks to provide this framework and apply it to a broad range of globally applicable SCS measures. The novelty of the approach therefore lies in the combination of (a) a broad initial scope; (b) the systematic selection and categorisation of a subset of specific measures; and (c) a multidisciplinary discussion of the pathways and barriers towards practical implementation of these measures.

## 2 DEFINING A FRAMEWORK FOR SCS MEASURE ASSESSMENT

Soil organic carbon (SOC) stock change is the difference between addition of organic C (typically as plant residue) and losses via harvested biomass and respiration (Paustian et al., 2016). While the soil C stock of land is often lowered by conversion to agriculture (Paustian et al., 2016; Six et al., 2002), once soil is under agricultural use, pathways to maximise sequestration of organic carbon can be categorised as follows:

1. Optimising crop primary productivity, particularly belowground (root) growth, and ensure the retention of this organic matter in the cropping system (increasing C inputs).
2. Adding C produced outside the cropping system (increasing C inputs).
3. Integrating additional biomass producers within the cropping system (increasing C inputs).
4. Minimising atmospheric release of CO_2_ from microbial mineralisation by reducing soil disturbance and managing soil physical properties (reducing C losses).
5. Minimising deliberate removal of C from the system or lateral transport of C via erosion processes (reducing C losses).

A long list of potential measures with the potential to deliver one or more of these outcomes was defined based on the review by Macleod, Eory, Gruère, and Lankoski (2015). These measures were reviewed by a panel of three experts and independently assessed against the following criteria:

1. Is the specified measure likely to lead to a significant increase in soil C storage?
2. What is the expert's confidence in the GHG abatement potential of the specified measure (including the ability of available modelling approaches to reliably quantify this potential)?
3. Is it likely that significant uptake, in addition to the business‐as‐usual scenario, could be achieved via policy?

**Figure 1:**
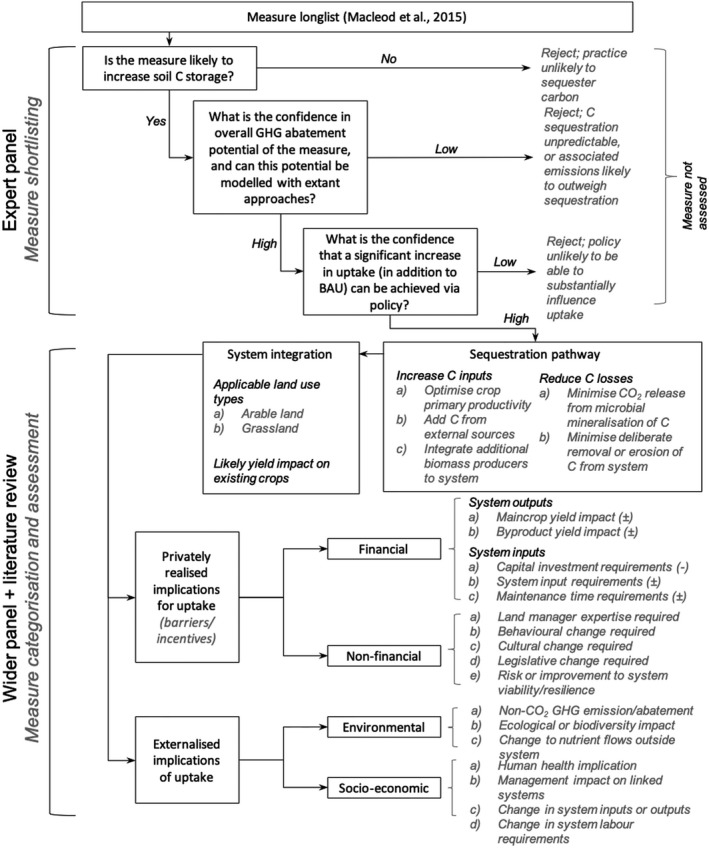
Systematic approach to selection and assessment of soil carbon sequestration measures followed for this analysis

This system allowed for sequential refinement of the long list into a shortlist of measures meeting the above criteria, with measures rejected at each stage (Figure 1). Following shortlisting, a framework, illustrated by Figure 1, was defined against which the measures could be categorised and assessed.

## 3 SELECTION AND ASSESSMENT OF SCS MEASURES

**Figure 2:**
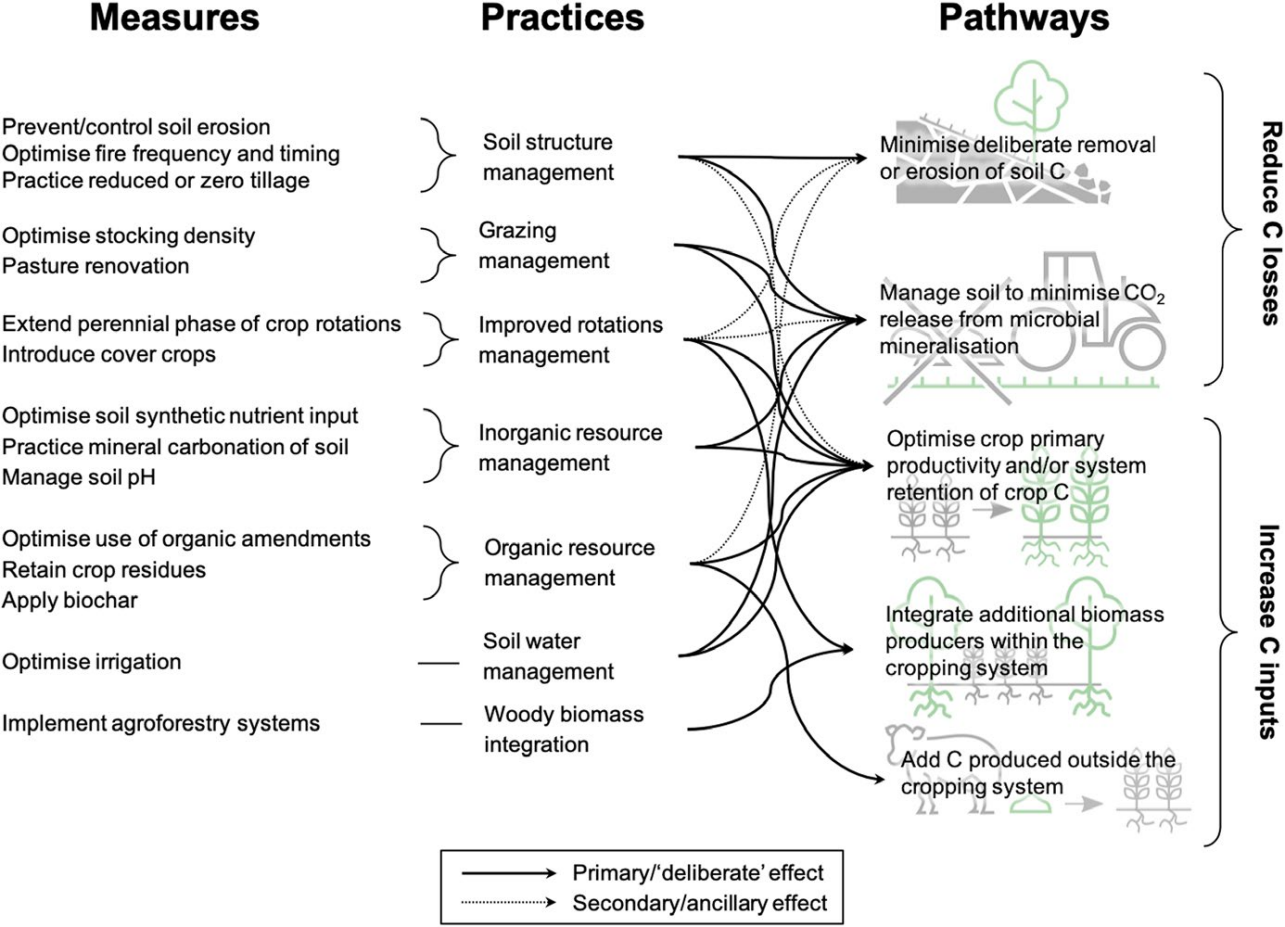
Results of the shortlisting and categorisation process for the selected SCS measures. Attribution of practices to pathways is expanded in Sections 3.1, 3.1.1, 3.1.2, 3.1.3, 3.2, 3.2.1, 3.2.2, 3.3, 3.3.1, 3.3.2, 3.4, 3.4.1, 3.4.2, 3.4.3, 3.5, 3.5.1, 3.5.2, 3.5.3, 3.6, 3.6.1, 3.7

Following shortlisting via the selection process defined in Figure 1, a group of 21 SCS measures, deemed to have technical potential according to these criteria, were selected. Based on further literature review focused around each shortlisted measure, these measures were sorted into categories representing consistent types of management practice, and further categorised according to the SCS pathway(s) relevant to each practice (Figure 2).

Whil the pathways defined can be attributed to specific measures, the categorisation of these measures into similar management practices lead to similar pathway attribution for each practice group, allowing the generalisation of pathways across practices as shown in Figure 2. These pathways were further attributed to specific measures, and the private and externalised impacts (as defined in the framework in Figure 1) were assigned to each measure based on the extant literature (Table 1).

**Table 1:** Defined soil carbon sequestration (SCS) measures by category, including estimates of applicability by land category, yield response, nature of private barriers and incentives and externalised impacts

Practice	Measure	Pathway(s)	Applicable land uses		Likely yield response	Private barriers and incentives		Externalised impacts	
			Crop production	Livestock production		Financial	Non‐financial	Environmental	Socio‐economic
Soil structure management	Prevent or control soil erosion	PP, MR	×	×	+	**C, M;** *Y*, *I*	**Ex;** *Re*	Nu	Ag
	Optimise fire frequency and timing	PP, MM	×	×	±	**M, Y**; *Y*	**Ex, Ri, Be, Po**	GG, *Eco*	He
	Practise reduced or zero tillage	MM	×	×	±	**C, I;** Y; *M*, *I*	**Ri:** *Re*	GG	
Grazing land management	Optimise stocking density	PP, MM		×	±	**Y, M**; *Y*	**Ex, Cu;** *Re*	*GG*, *Eco*, Nu	La
	Renovate unimproved pasture	PP		×	+	**M, I, C**; *Y*	**Be, Inf;** *Re*	*GG*, Eco	In
Improved rotation management	Extend perennial phase of crop rotations	PP, MM, MR	×		+	**Y**			Out
	Implement cover cropping	AB, MR	×		+	**I, M**; Y; *I*	**Ri;** *Re*	Nu	In
Inorganic resource management	Optimise soil synthetic nutrient input	PP	×	×	+	**I**; *Y*	**Ex, Be, Inf;** *Re*	**GG**	**He**, In
	Practise mineral carbonation of soil	MM	×	×	±	**I, M**; *I*, *Y*	**Ri, Ex, Inf**	GG, Nu, *Eco*	He, In, La
	Manage soil pH	PP, MM	×	×	+	**I, M**; *Y*, *I*	**Ex, Be**	**GHG**, Nu, Eco	In, La
Organic resource management	Optimise use of organic amendments	AC, PP, MR	×	×	+	**M, B, C**; *Y*, *I*	**Ex, Inf;** *Re*	GG, Nu	**He**, Ag, In, Out
	Retain crop residues	MR	×		+	**B, C, M**; *I*	**Be, Re**	GHG, *Eco*	In, Out
	Apply biochar	AC, PP	×		+	**B, I, M**; *Y*, *I*	**Ri, Po, Be, Ex, Inf;** *Re*	*GG*, **Al**, Nu	In, La
Soil water management	Optimise irrigation	PP, MM	×	×	+	**C, M**; *Y*	**Ex, Be**	**GG,** Nu	In, He
Woody biomass integration	Implement agroforestry systems	AB	×	×	+	**C, I, M**; Y; *B*	**Ri, Be;** *Re*	*Eco*	In, Out

Note

**All columns** *.* Bold text = barrier or negative impact, italicised text = incentive or positive impact, normal text = direction not specified, bidirectional or not applicable.

**Pathways**. [PP] = maximise primary productivity of existing crops, [MM] = manage soil properties to minimise C mineralisation, [MR] = minimise deliberate removal or erosion of C, [AC] = add external C to system or avoid C removals, [AB] = include additional biomass producers in system.

**Yield response**. [+] = positive yield response, [−] = negative yield response, [±] = bidirectional (context‐specific) response, [*n*] = neutral response.

**Private financial barriers/incentives**. [Y] = main crop yield (increase/loss), [B] = by‐product yield (increase/loss), [C] = capital investment required to implement measure, [I]  = agrochemical input (increase/offset), [M] = maintenance/time cost (increase/offset).

**Private non‐financial barriers/incentives**. [Ex] = land manager expertise required to implement measure, [Be] = behavioural barrier, that is, measure likely to require substantial change to habitual behaviour, [Ri] = perceived risk to production system viability associated with implementing measure, [Cu] = cultural barrier, [Po] = potential policy‐based or legislative barrier to implementing measure, [Re] = agroecosystem resilience affected by implementation.

**Environmental externalities**. [GG] = GHG emission or reduction (in addition to SCS), [Nu] = change to agroecosystem nutrient flows, [Al] = albedo effect on affected soils, [Eco] = ecological or biodiversity impact on connected ecosystems.

**Socio‐economic externalities**. [He] = human health implication, [Ag] = management impact for linked agroecosystems, [In] = qualitative change in system input demand, [Out] = qualitative change in supply of system outputs, [La] = change in labour demand for production system.

The remainder of this section maps to the framework of Table 1 and comprises the results of the review process for each practice from in terms of (a) the technical biophysical context and pathways to SCS; (b) private barriers and incentives to implementation of measures by land managers; and (c) externalised impacts of implementation. Where it is possible to quantify or attribute a direction of change to an impact, this is described based on the extant literature; however, many impacts are either non‐directional in nature or context‐specific dependent on the agricultural systems or baselines to which they are applied.

### 3.1 Soil structure management

Soil structure management comprises measures which have the main goal of improving soil physical structure and preventing excessive lateral transport or mineralisation of existing soil C fractions. While lateral transport of C reduces only local stocks by definition, improving local soil C storage in this way may also provide increased availability of labile C fractions, the mineralisation of which provides nutrients for plant growth (Chenu et al., 2019); as such, these measures may also indirectly increase SOC inputs via increased primary productivity.

#### 3.1.1 Prevent or control soil erosion

##### Sequestration pathways (primary productivity, minimised removal)

The role of erosion is an important uncertainty in the quantification of the global potential of soils to sequester C (Doetterl et al., 2016). Agricultural activities have accelerated erosion processes; global SOC erosion is estimated between 0.3 and 0.5 Gt C/year (Chappell, Baldock, & Sanderman, 2015; Doetterl et al., 2016). Erosion and deposition of SOC concentrate it in depositional sites, without directly changing the net regional C balance, though alters the biological factors which drive the mineralisation of SOC; this may result in a net overall change in stocks (Doetterl et al., 2016; Gregorich, Greer, Anderson, & Liang, 1998; Lugato et al., 2018; Luo, Wang, Sun, Smith, & Probert, 2011). However, the most tangible SOC impact of erosion is through loss of primary productivity, reducing organic inputs (Gregorich et al., 1998).

##### Private financial barriers and incentives (capital, maintenance; yield, inputs)

Permanent or semi‐permanent measures are likely to require significant capital investment (Posthumus, Deeks, Rickson, & Quinton, 2015) Non‐permanent erosion control measures (e.g. contour cropping) may incur a time cost or investment in specialist equipment (Frelih‐Larsen et al., 2014). Yield improvements are likely as soil retention improves (Dorren & Rey, 2004; Marques Da Silva & Alexandre, 2004), and this may also reduce costs associated with agrochemical and irrigation inputs (Stevens et al., 2009).

##### Private non‐financial barriers and incentives (expertise; resilience)

Measures are likely to require local expertise to select, design and implement (Frelih‐Larsen et al., 2014). Agroecosystem resilience to extreme weather is likely to improve as a result (Lal, 2003).

##### Environmental externalities (nutrients)

Nutrient losses from system to catchment are likely to be reduced by erosion control measures, reducing water pollution (Chappell et al., 2015; Doetterl et al., 2016).

##### Socio‐economic externalities (agroecosystem)

Agroecosystems in lower catchment areas may lose fertile sediments transported from upper landscape positions (Fiener, Dlugoß, & Van Oost, 2015).

#### 3.1.2 Optimise fire frequency and timing

##### Sequestration pathways (primary productivity, minimalised mineralisation)

In arid regions, rangeland burning is used to control bush encroachment (Lehmann et al., 2006; Lorenz & Lal, 2014; Vågen et al., 2005), to improve the quality of grazing land (Snyman, 2004) and to increase plant species diversity (Furley, Rees, Ryan, & Saiz, 2008). It is also used to manage heather on upland temperate soils (Yallop, Clutterbuck, & Thacker, 2012). Burning of land increases C inputs to the soil via char, unburned surface litter and un‐combusted root matter (Knicker, 2007), while the heat may precipitate thermal decomposition of SOC. Fire may also affect soil physical properties, destabilising soil structure and increasing bulk density. Seasonal timing of burns is critical in terms of the impact on SOC (Fynn, Haynes, & O'Connor, 2003; Hunt, 2014; Vågen et al., 2005), and response is highly context‐specific (Hunt, 2014; Knicker, 2007); optimisation may mean (a) wildfire control; (b) increase or decrease in frequency of deliberate burns; or (c) alteration to timing of burn to reduce intensity.

##### Private financial barriers and incentives (maintenance, yield; yield)

Reduction in fire frequency may increase costs such as control of bush encroachment (Lorenz & Lal, 2014), which may reduce livestock grazing potential (Vågen et al., 2005). However, optimisation may allow heavier grazing practices without damage to SOC stocks (McSherry & Ritchie, 2013).

##### Private non‐financial barriers (expertise, risk, behavioural, policy)

Availability of expertise regarding optimal practice may challenge implementation. An additional barrier may be land manager perception of risk (e.g. fear of yield or income losses) as well as resistance to behavioural change. Existing regional and national policy may restrict land manager control over burning regimes (Biggs & Potgieter, 1999).

##### Environmental externalities (GHG, ecosystem)

Changes to fire regimes will impact direct CO_2_ release (Hunt, 2014), as well as non‐CO_2_ climate forcers (e.g. black carbon) and air pollutants. While the CO_2_ is taken up as vegetation regrows, timescales vary from a few years (e.g. in savannas) to 100s of years (e.g. peatlands; Joosten, 2010). Ecosystem ecology may be closely linked with fire frequency (e.g. Bond & Keeley, 2005), so restoration of natural regimes may have positive ecological impacts. Changes to resulting air pollutant load may also have ecological impacts (Bowman & Johnston, 2005).

##### Socio‐economic externalities (health)

Uncontrolled fires present a danger to local populations, and all burns cause pollutant emissions with associated human health impacts (Bowman & Johnston, 2005).

#### 3.1.3 Practice reduced or zero tillage

##### Sequestration pathways (minimised mineralisation)

Reduced tillage and no‐till systems preserve aggregates which physically protect C from mineralisation (Merante et al., 2017; West & Post, 2002). SCS response is context‐specific; many studies (e.g. van Kessel et al., 2013; Paustian, Six, Elliott, & Hunt, 2000; Six et al., 2004) show a positive effect, while others show a negative or neutral response (Álvaro‐Fuentes, López Sánchez, Cantero‐Martínez, & Arrúe Ugarte, 2008; Christopher, Lal, & Mishra, 2009; Sisti et al., 2004). Soil texture is likely to influence strongly efficacy of this practice (Gaiser, Abdel‐Razek, & Bakara, 2009).

##### Private financial barriers and incentives (capital, inputs; yield; maintenance, inputs)

Capital investment in new equipment may be necessary (Posthumus et al., 2015). Additional pesticides, particularly herbicides, may be required to remove weeds, pests and previous crops where no‐till is adopted (Beehler, Fry, Negassa, & Kravchenko, 2017; Gaiser, Stahr, Billen, & Mohammad, 2008; Maillard, McConkey, St. Luce, Angers, & Fan, 2018). The measure has potential to increase crop yield, though losses are also possible, particularly in wetter regions (Ogle, Swan, & Paustian, 2012; Pittelkow et al., 2015). No‐till reduces fuel and time costs associated with cultivation, germination success in dry soils may be enhanced and irrigation requirements may reduce (Pareja‐Sánchez et al., 2017; Schlegel et al., 2016).

##### Private non‐financial barriers (risk; resilience)

This practice may, correctly or not, be perceived as likely to induce yield loss (Grandy, Robertson, & Thelen, 2006); agronomic challenges (e.g. potential for weed and pest build‐up) may also impact perceptions. In contrast, bare fallow reduction and increased aggregate stability will contribute erosion resilience (Marques Da Silva & Alexandre, 2004; Pittelkow et al., 2015).

##### Environmental externalities (GHG)

Reduced or no‐till uses less energy per unit area, reducing GHG emissions from cultivation (Williams, Audsley, & Sandars, 2010). In some circumstances, reduced tillage can be associated with increased N_2_O emissions (Powlson et al., 2014).

### 3.2 Grazing land management

Measures collated under this management practice represent those which specifically apply to land under direct livestock production. These measures therefore involve either directly managing livestock or managing the grass sward, such that C sequestration is optimised under grazing. The net effect of these measures is to improve either overall primary productivity or its retention in grassland soils.

#### 3.2.1 Optimise stocking density

##### Sequestration pathways (primary productivity, minimised mineralisation)

Optimised intensity grazing maximises primary productivity and proportionally increases belowground fractions (Garnett et al., 2017; Reeder & Schuman, 2002; Wienhold, Hendrickson, & Karn, 2001). Optimal intensity is context‐specific; some grazing may increase belowground C, while overgrazing results in mineralisation of existing SOC and decreases C returns; this response is metered by factors including primary productivity, livestock type, soil texture, initial SOC content and sward composition (Abdalla et al., 2018; Lu et al., 2017; McSherry & Ritchie, 2013; Stockmann et al., 2013; Zhou, Zhou, He, et al., 2017). In particular, the growth form of the dominant grass species types (C_3_ vs. C_4_) may impact the direction of grazing response. Livestock manure deposition may also improve the transfer of OC to stable pools (McSherry & Ritchie, 2013; Rutledge et al., 2017a, 2017b).

##### Private financial barriers and incentives (yield, maintenance; yield)

Optimal stocking density should give high sustainable yield, though may incur short‐term losses (McSherry & Ritchie, 2013). If optimisation increases system complexity (e.g. rotational or mob grazing), time costs may be incurred (Waters, Orgill, Melville, Toole, & Smith, 2017).

##### Private non‐financial barriers (expertise, cultural; resilience)

Effective optimisation requires local expertise. In cultures where livestock ownership contributes to perceived wealth (e.g. sub‐Saharan Africa), reduction may be difficult to incentivise (Oba, Stenseth, & Lusigi, 2000). However, implementation should benefit agroecosystem resilience to pests, erosion processes and weather events (Keim, Lopez, & Balocchi, 2015).

##### Environmental externalities (GHG, ecosystem, nutrients)

Optimisation of stocking density will impact availability and quality of forage, and hence impact CH_4_ from enteric fermentation, and GHGs and nutrient leaching from manure (Dong, Mangino, & McAllister, 2006; de Klein et al., 2006). Grazing pressure precipitates direct and indirect biodiversity impacts as a result of changes to sward composition (Bruinenberg, Valk, Korevaar, & Struik, 2002; Derner, Boutton, & Briske, 2006; Frank, Tanaka, Hofmann, & Follett, 1995).

##### Socio‐economic externalities (labour)

A change in herd size or grazing extent may impact system labour requirements (Dillon, Roche, Shalloo, & Horan, 2005).

#### 3.2.2 Renovate unimproved pasture

##### Sequestration pathways (primary productivity)

Pasture renovation is typically undertaken to improve the yield and nutritional quality of grazing (Bruinenberg et al., 2002; Frame & Laidlaw, 2011). Soil C input is increased though higher primary productivity, though soil disturbances and interruption of C inputs may result from removal of the old sward (Mudge et al., 2011; Rutledge et al., 2017a, 2017b). Optimal implementation may include deep‐rooting grasses, such as *Brachiaria* spp., which have the potential to enhance SCS by improving belowground inputs (Amézquita, Murgueitio, Ibrahim, & Ramírez, 2008; Costa et al., 2016; Fisher et al., 1994; Stahl et al., 2017). Increased sward biodiversity has also been shown to drive SOC accumulation (Cong et al., 2014; De Deyn et al., 2009; Mueller, Tilman, Fornara, & Hobbie, 2013; Rutledge et al., 2017a; Tilman, Wedin, & Knops, 1996).

##### Private financial barriers and incentives (maintenance, capital, inputs; yield)

Costs are likely to stem from equipment, maintenance and input requirements (Bruinenberg et al., 2002; Frame & Laidlaw, 2011). Increased stocking rates and feed conversion of grazing animals are likely (Bruinenberg et al., 2002).

##### Private non‐financial barriers (behavioural, infrastructure; resilience)

Required change to habitual practices may present a behavioural barrier. For developing regions, access to the requisite expertise, capital items and inputs may preclude implementation (e.g. Cardoso et al., 2016). Optimal implementation may increase system resilience to climate change, disease and pests (Barker, 1990; McSherry & Ritchie, 2013).

##### Environmental externalities (GHG, ecosystem)

Pasture renovation is likely to increase agrochemical‐related emissions, but reduce enteric CH_4_ from livestock (Dong et al., 2006; de Klein et al., 2006). Alterations to sward species composition will precipitate direct and indirect biodiversity impacts (Bruinenberg et al., 2002; Meek et al., 2002).

##### Socio‐economic externalities (input demand)

This measure will create local demand for additional agricultural inputs and agrochemicals (e.g. Cardoso et al., 2016).

### 3.3 Improved rotation management

Measures grouped under this practice category focus on improving the management of crop rotations to either (a) increase the retention of biomass by the cropping system or (b) integrate additional biomass producers into the existing rotations. Both strategies tend to increase long‐term ground cover, with the ancillary effects of reducing soil disturbance and minimising erosion.

#### 3.3.1 Extend the perennial phase of crop rotations

##### Sequestration pathways (primary productivity, minimised mineralisation, minimised removal)

Diversification of arable cropping systems with perennial plants, such as grass leys, serves to increase the quantity and continuity of belowground residue returned to the soil, and can support microbial activity and diversity (Fu, Wang, Sainju, & Liu, 2017; West & Post, 2002). Mineralisation of existing stocks due to disturbance will also be reduced (Gentile, Martino, & Entz, 2005; Johnston, Poulton, Coleman, Macdonald, & White, 2017; Prade, Kätterer, & Björnsson, 2017). Other perennial crops introduced into arable rotations may include woody (Don et al., 2012; Heller, Keoleian, & Volk, 2003) or non‐woody (Sainju, Singh, & Singh, 2017) biomass crops for bioenergy.

##### Private financial barriers and incentives (yield)

The majority of studies comparing to arable‐only rotations find a net reduction in arable production (Johnston et al., 2017; Persson, Bergkvist, & Kätterer, 2008; Prade et al., 2017), though annual yield may increase in the long term.

##### Socio‐economic externalities (output supply)

System establishment is likely to reduce arable outputs, and increase those derived from the perennial crop (e.g. Heller et al., 2003; Prade et al., 2017).

#### 3.3.2 Implement cover cropping

##### Sequestration pathways (additional biomass, minimised removal)

Cover crops are grown primarily to maintain soil cover during winter fallow periods (Ruis & Blanco‐Canqui, 2017), and may serve to prevent N leaching (Cicek, Martens, Bamford, & Entz, 2015) or provide nutrition to the main crop (Alliaume, Rossing, Tittonell, Jorge, & Dogliotti, 2014; Dabney et al., 2010); these functions can be combined, as in crucifer–legume mix cover crops (Couëdel, Alletto, Tribouillois, & Justes, 2018). Year‐round soil cover serves to prevent erosion (De Baets, Poesen, Meersmans, & Serlet, 2011), decrease N leaching (Blombäck, Eckersten, Lewan, & Aronsson, 2003) and increase main crop productivity (Lal, 2004). Poeplau and Don (2015) showed that cover cropping can also minimise SOC loss between rotations; systems avoiding or reducing fallow have been demonstrated to increase soil C stocks independently of other factors (Gentile et al., 2005; Goglio, Bonari, & Mazzoncini, 2012; Goglio, Smith, Grant, et al., 2018).

##### Private financial barriers and incentives (inputs, maintenance; yield; inputs)

Establishment of this measure will induce additional input and time costs. Main yield effects are context specific (Poeplau & Don, 2015). The cover crop may provide by‐products (e.g. green manure) to the main crop (Ruis & Blanco‐Canqui, 2017), and use of some agrochemicals may also reduce under some cover crop rotations (Snapp et al., 2005).

##### Private non‐financial barriers (risk; resilience)

Risk of yield loss or negative pest control impacts may disincentivise implementation (Garcia et al., 2018). Soil erosion resistance should improve with reduction of bare fallow (Van den Putte, Govers, Diels, Gillijns, & Demuzere, 2010).

##### Environmental externalities (GHG, ecosystem)

Cover cropping is demonstrated to reduce N_2_O emissions (Eory et al., 2015; Pellerin et al., 2013). Pest control requirements are likely to change, though this response is bidirectional with positive (Snapp et al., 2005) and negative (Posthumus et al., 2015) elements.

##### Socio‐economic externalities (input demand)

Establishment of the cover crop will require inputs (Garcia et al., 2018), and may offset demand for agrochemicals required by the main crop (Ruis & Blanco‐Canqui, 2017).

### 3.4 Inorganic resource management

These measures employ inorganic resources to modify soil properties, serving either to improve nutrient availability to crops, increase primary productivity or reduce the likelihood of CO_2_ release to the atmosphere via microbial mineralisation. Mineral carbonation stands distinct from all other measures assessed in this study in that it provides a permanent soil‐based sink for mineralised organic C (Beerling et al., 2018).

#### 3.4.1 Optimise soil synthetic nutrient input

##### Sequestration pathways (primary productivity)

Stoichiometric limitations to SOC accumulation are present in many agroecosystems (Kirkby et al., 2013; Van Groenigen et al., 2017); optimum SCS requires N availability in addition to that required for optimal crop production (Kirkby et al., 2014). Optimisation of nutrient (particularly N) input therefore has potential to maximise yield and SOC accumulation in arable systems (Chaudhary, Dheri, & Brar, 2017; Jokubauskaite, Karčauskienė, Slepetiene, Repsiene, & Amaleviciute, 2016; Lu et al., 2009; Yang, Zhao, Huang, & Lv, 2015). Most studies find that mixing synthetic and organic amendments optimises SCS, and some (e.g. Su, Wang, Suo, Zhang, & Du, 2006) report negative SCS in the absence of organic fertiliser.

##### Private financial barriers and incentives (inputs; yield)

Fertiliser costs will increase, though yield will increase substantially in many regions (Mueller et al., 2012). At optimal SCS, some nutrients remain sequestered in SOC compounds rather than plant matter (Kirkby et al., 2014), resulting in a cost not compensated by yield increase.

##### Private non‐financial barriers (expertise, behaviour, infrastructure; resilience)

Land manager expertise will be required, and reluctance to rely on purchased inputs may be a disincentive (Cook & Ma, 2014). Fertiliser availability may present an infrastructure barrier in developing nations. This measure should increase agroecosystem resilience (Goglio et al., 2012, 2014; Shehzadi, Shah, & Mohammad, 2017).

##### Environmental externalities (GHG, nutrients)

Greenhouse gas emissions associated with production and application of synthetic fertiliser are likely to increase (Goglio et al., 2012, 2014; Schlesinger, 2010). This measure will alter nutrient flows within and beyond the system (Kirkby et al., 2013).

##### Socio‐economic externalities (health, input demand)

Negative health impacts may result from increased fertiliser use (e.g. Brainerd & Menon, 2014). The measure is also likely to increase local demand for agrochemical inputs (Mueller et al., 2012).

#### 3.4.2 Practice mineral carbonation of soil

##### Sequestration pathways (minimised mineralisation)

Following microbial mineralisation, a proportion of organic carbon in soils becomes fixed as pedogenic carbonates (Cerling, 1984). Amendment of soils with weatherable calcium sources, such as calcium‐bearing silicate rocks, and the consequent formation of calcium carbonates provide a permanent sink for mineralised organic C (Beerling et al., 2018; Manning, Renforth, Lopez‐Capel, Robertson, & Ghazireh, 2013).

##### Private financial barriers and incentives (inputs, maintenance; inputs, yield)

Purchase of material comminuted to maximise GGR is required, ad application may incur time costs (Renforth, 2012). Rigorous determinations of yield benefits of crushed basaltic rocks are few (Beerling et al., 2018), but recent studies show some successes (e.g. de Fátima Tavares, Carvalho, Camargo, Fátima Pereira, & Cardoso, 2018).

##### Private non‐financial barriers (risk, expertise, infrastructure)

Risk of yield non‐response or health impacts may disincentivise uptake (Pidgeon & Spence, 2017). Lack of a broad research base may present a knowledge barrier (Beerling et al., 2018). Global application depends on the ability to source calcium‐bearing silicate rocks and to deliver these in appropriate form to farms for application.

##### Environmental externalities (GHG, nutrients, ecosystem)

Mining, grinding and spreading of rock may have negative ecological impacts on affected areas, and may lead to GHG emissions related to energy use; if sourced as a by‐product, impacts are minimised, though production would have to increase 10‐fold to reach GGR scenarios suggested by Beerling et al. (2018). If fertiliser use is reduced as a result of crushed rock application, net GHG emissions may be reduced. Losses of CaCO_3_ to the system catchment are likely; these may ultimately act to increase ocean alkalinity and stimulate growth of calcareous organisms (Beerling et al., 2018).

##### Socio‐economic externalities (health, input demand, labour)

Implementation of this measure is likely to increase demand for crushed rock and may reduce fertiliser demand (Beerling et al., 2018). Quarrying and processing of these rocks is widespread, with associated human health impacts (e.g. dust inhalation) mostly well understood. System labour demands may be altered by implementation of this measure.

#### 3.4.3 Manage soil pH

##### Sequestration pathways (primary productivity, minimised mineralisation)

Optimising soil pH generally consists of reducing soil acidity through application of alkaline calcium or magnesium carbonates or oxides, known as lime, or reducing sodicity via gypsum applications (Hamilton, Kurzman, Arango, Jin, & Robertson, 2007). Calcium carbonate‐rich soils provide free calcium, which binds with OM to form complex aggregates, providing physical protection from microbial decomposition (Tu, He, Lu, Luo, & Smith, 2018). Optimal pH improves soil nutrient availability, increasing primary productivity and OM input to soil (Ahmad, Singh, Dijkstra, & Dalal, 2013; Holland, White, Glendining, Goulding, & McGrath, 2019). However, liming also increases C and N mineralisation (Chenu et al., 2019; Paradelo, Virto, & Chenu, 2015), accelerating losses as well as increasing inputs and making net SCS response context‐specific.

##### Private financial barriers and incentives (inputs, maintenance; yield, inputs)

Lime or gypsum must be purchased to implement. Yield improvements may offset this, though upfront cash cost may be prohibitive in developing nations (Mitchell et al., 2003), and application will incur time costs. Optimisation of this measure may reduce requirements for other agrochemical inputs (Fornara et al., 2011).

##### Private non‐financial barriers (expertise, behavioural)

Expertise is required to optimise application. Resistance to becoming reliant on externally priced inputs disincentivise uptake (Mitchell et al., 2003).

##### Environmental externalities (GHG, nutrients, ecosystem)

Lime application releases CO_2_ (de Klein et al., 2006), but microbial communities also respond by increasing the N_2_/N_2_O ratio during denitrification, potentially reducing N_2_O emissions (Goulding, 2016). Extraction, transportation and application of lime will affect nutrient flows and energy‐related CO_2_ emissions. If demand for lime increases, increased extraction rates may cause ecological impacts at extraction sites (Salomons, 1995).

##### Socio‐economic externalities (input demand, labour)

Increased application rates will create local demand. Smaller scale extraction (e.g. Mitchell et al., 2003) may involve in‐system processing, which will alter labour requirements.

### 3.5 Organic resource management

These measures transfer existing organic carbon to the soil pool. This in itself is soil C storage (Chenu et al., 2019), but where this transfer to the soil C pool (vs. other uses) increases long‐term C removal from the atmosphere, it represents net sequestration. Organic amendments may also improve crop primary productivity via increased nutrient availability and labile C fractions; this represents a secondary pathway by which this measure can influence net atmospheric C removal.

#### 3.5.1 Optimise use of organic amendments

##### Sequestration pathways (additional carbon, primary productivity, minimised removal)

Optimal application of organic fertilisers has potential to contribute to soil carbon storage in croplands and grasslands (Chaudhary et al., 2017; Jokubauskaite et al., 2016; Shahid et al., 2017; Wang, Hu, et al., 2015; Yang et al., 2015). Organic manure is commonly applied and effective, though green manures are also important (Wang, Yang, et al., 2015). Both improve agroecosystem productivity through returning organic C to the soil in addition to other nutrients, improving soil structure and water retention and reducing erodibility (Brady & Weil, 2002; Shehzadi et al., 2017). The alternative fate of the organic material used is important; net sequestration will occur only where (a) the organic amendments are produced by or for, rather than repurposed to, the agroecosystem; or (b) where the C in existing amendments would otherwise be more rapidly lost to the atmosphere, such as through burning (e.g. Sandars et al., 2003). The latter may also be possible to achieve via reapportionment of resources to land with lower C stocks; organic material tends to be applied on grazing land (Chaudhary et al., 2017; Sainju, Senwo, Nyakatawa, Tazisong, & Reddy, 2008), which typically has a higher C equilibrium than croplands (Verchot et al., 2006).

##### Private financial barriers and incentives (maintenance, by‐products, capital; yield, inputs)

Organic fertiliser application has labour and time costs in comparison to equivalent synthetic fertiliser (Yang et al., 2015), and costs may result if amendments are normally sold or otherwise utilised (e.g. Williams, Leinonen, & Kyriazakis, 2016). Optimisation should increase yields, or may offset requirements for more expensive inputs (e.g. synthetic NPK). Increased soil quality may reduce other costs (e.g. irrigation, agrochemical inputs; Shehzadi et al., 2017).

##### Private non‐financial barriers (expertise, infrastructure; resilience)

Land manager expertise is required to optimise application rates. Transport of organic amendments requires an effective and low‐cost transport network, which may be a barrier in developing nations. Increased soil aggregative stability will improve agroecosystem resilience to erosion and extreme weather (Shehzadi et al., 2017).

##### Environmental externalities (GHG, nutrients)

Manure may be burned for fuel or electricity; reapportioning risks ‘leakage’ if higher emitting processes fill this demand (Williams et al., 2016). Emissions from manure storage and application may change (de Klein et al., 2006; Saggar, 2010), and emissions from synthetic fertiliser production may be indirectly impacted. Nutrient flows to and from the system are likely to be altered (Shehzadi et al., 2017).

##### Socio‐economic externalities (health, agroecosystem, input demand, output supply)

Use of manure on human‐edible crops, and transfer of manure between systems, has associated human and animal health implications (Amoah, Drechsel, & Abaidoo, 2005; Liu et al., 2013). Local supply and demand for organic and synthetic fertilisers will be affected.

#### 3.5.2 Retain crop residues

##### Sequestration pathways (minimised removal)

Removal of crop residues for use as animal feed, bedding, fuel, industrial feedstock and building material is common; removal of this organic carbon stock results in a loss of SOC (Ruis & Blanco‐Canqui, 2017; Smith, 2012). Retention of residues is therefore likely to induce positive changes in SOC (Wang, Yang, et al., 2015) and crop yield (Hu et al., 2016). Residue incorporation is associated with increased N_2_O and CH_4_ emissions (Hu et al., 2016; de Klein et al., 2006; Koga & Tajima, 2011), but overall GHG emissions can be reduced by use of appropriate tillage (Ball et al., 2014; Tellez‐Rio et al., 2017).

##### Private financial barriers and incentives (by‐products, capital, maintenance; inputs)

Residues will be rendered unavailable for other uses by this measure. Capital investment in new equipment, and a time cost may be necessary to process or reincorporate residues (Garcia et al., 2018). Fertiliser costs may be partially offset by nutrients from retained residues (e.g. Prade et al., 2017).

##### Private non‐financial barriers (behaviour, resilience)

Given many alternative uses for residues, overcoming habitual behaviour may be a significant barrier to implementation. Pest and disease control is impacted by residue management, and returning crop residues may negatively impact agroecosystem resilience (Bailey & Lazarovits, 2003).

##### Environmental externalities (GHG, ecosystem)

Incorporation of residues may incur direct N_2_O and CH_4_ emissions (de Klein et al., 2006), though may offset emissions from fertiliser. There is also potential for emissions ‘leakage’ if reallocation precludes residue availability for other GHG‐offsetting activities (e.g. biofuel production; Kim & Dale, 2004). Biodiversity of the microbial community is likely to be improved by residue retention (Govaerts et al., 2007; Turmel, Speratti, Baudron, Verhulst, & Govaerts, 2015).

##### Socio‐economic externalities (input demand, output supply)

Demand for substitute materials to fulfil foregone applications (e.g. fuels, livestock feeds), or reduction the supply of residues for off‐system uses, is likely.

#### 3.5.3 Apply biochar

##### Sequestration pathways (additional carbon, primary productivity)

Biochar is pyrogenic organic matter produced by a high‐temperature, low‐oxygen conversion of biomass. Biochar contributes to SCS owing to its high C content and high recalcitrance (Lehmann, 2007). In principal, this offers an unlimited sink for C in soil, as well as more permanent changes in other soil properties. General positive effects on primary productivity (Jeffery et al., 2017) may be attributed to increased soil pH, and nutrient and moisture availability. A small proportion of C in biochar is much less stable than the rest, and the addition of labile C can induce a ‘priming’ effect where microbial biomass is increased over the short term (Kuzyakov, 2010; Kuzyakov, Friedel, & Stahr, 2000). This effect is highly context‐specific (Kuzyakov, 2010; Kuzyakov et al., 2000; van der Wal & de Boer, 2017; Zimmerman, Gao, & Ahn, 2011), with reported examples of positive (Wardle, Nilsson, & Zackrisson, 2008), neutral (Novak et al., 2010) and negative (Weng et al., 2017) priming effects on soil C stocks. Regardless of short‐term impact, long‐term SOC impact of biochar amendment is positive (Liu et al., 2016; Maestrini, Nannipieri, & Abiven, 2015; Wang, Xiong, & Kuzyakov, 2016; Zhou, Zhou, Zhang, et al., 2017; Zhou, Zhang, et al., 2017).

##### Private financial barriers and incentives (by‐products, inputs, maintenance; yield, inputs)

Biochar must be purchased or produced, with variable cost depending on source material, labour and processing. Agricultural by‐products (e.g. residues) may be utilised (Jones, Rousk, Edwards‐Jones, DeLuca, & Murphy, 2012), though this precludes their sale or use elsewhere. Positive impacts on pH, passive buffering, soil water, soil microbial community and soil nutrient dynamics give potential for yield improvements (Joseph et al., 2013; Qian et al., 2014; Xu & Chan, 2012), and integration of biochar into existing agricultural inputs may improve efficiency of nutrient delivery (Xu & Chan, 2012).

##### Private non‐financial barriers (risk, policy, expertise, behaviour, infrastructure; resilience)

Barriers to uptake may include resistance to increased system complexity, perceived risk of non‐response and reluctance to rely on purchased inputs; supply chain infrastructure may also present a challenge (Lehmann et al., 2006; Meyer, Glaser, & Quicker, 2011). The regulatory position regarding the use of biochar may take time to resolve. By contrast, biochar‐amended soil is likely to have greater aggregate stability and erosion resilience (Liang et al., 2014).

##### Environmental externalities (GHG, albedo, nutrients)

Except for wet feedstock, the energy required for biochar production can be recovered from the gases produced in pyrolysis (Lehmann, 2007). Application generally decreases N_2_O emissions (He et al., 2017; Schirrmann et al., 2017) and CH_4_ emissions in the case of flooded rice (Song, Pan, Zhang, Zhang, & Wang, 2016). Application of biochar can darken its soil, with the resultant reduction in albedo reducing the net GHG mitigation benefit by up to 22% (Meyer, Bright, Fischer, Schulz, & Glaser, 2012).

##### Socio‐economic externalities (input demand, labour)

Demand for biochar or raw materials will be created, and system labour requirements may change, particularly if biochar is produced on‐site.

### 3.6 Soil water management

#### 3.6.1 Optimise irrigation

##### Sequestration pathways (primary productivity, minimised mineralisation)

Optimal irrigation can improve SCS in water‐scarce systems by increasing primary productivity and OM input to the soil (Guo et al., 2017); increased SOC improves soil water holding and plant water use efficiency (Shehzadi et al., 2017), feeding back into the efficacy of irrigation practices and optimal management of soil moisture may also serve to inhibit microbial decomposition of SOC (Guo et al., 2017). Over‐irrigation may reduce SOC stocks through reduced plant investment in root systems, or increased microbial mineralisation from frequent wetting–drying cycles (Mudge et al., 2017).

##### Private financial barriers and incentives (capital, maintenance; yield)

Costs are likely to stem from investment in equipment, construction and system maintenance (e.g. Zhang et al., 2018). These range from on‐farm costs to collective structures such as dams, reservoirs or even a national grey water network (Haruvy, 1997). Water abstraction may be a direct cost. Crop yield and quality are likely to increase (Mudge et al., 2017; Zhang et al., 2018).

##### Private non‐financial barriers (expertise, behavioural)

Expertise is required to implement and optimise the system, and the required increase in complexity and maintenance may disincentivise uptake.

##### Environmental externalities (GHG, nutrients)

Irrigation may trigger denitrification and N_2_O emissions from soils (Saggar, 2010; Snyder, Bruulsema, Jensen, & Fixen, 2009), can exacerbate phosphate run‐off and nitrate leaching and may alter nutrient flows in the agroecosystem.

##### Socio‐economic externalities (input demand, health)

Where irrigation results in increased water demand, conflict may result between agriculture and direct human or industrial needs, given the finite supply of water resources (Vörösmarty, Green, Salisbury, & Lammers, 2000).

### 3.7 Woody biomass integration

#### 3.7.1 Implement agroforestry systems

##### **Sequestration pathways** (additional biomass)

Agroforestry refers to the practice of growing trees in crop or livestock systems; it encompasses several implementations and can be applied to intercropped systems (e.g. alley cropping), fallow management, wind or shelter belts and grazing (Nair, Nair, Mohan Kumar, & Showalter, 2010). For each, the resulting woody biomass inputs represent a key route to SCS (Lorenz & Lal, 2014); in addition to C sequestration in aboveground tree biomass, with ongoing transfer to the soil C pool, tree roots improve the quality and quantity of belowground C inputs, and recover nutrients and moisture from lower soil horizons (Lorenz & Lal, 2014). Overall agroecosystem primary productivity is likely to increase (Burgess & Rosati, 2018).

##### Private financial barriers and incentives (capital, inputs, maintenance; yield; by‐products)

Capital investment is required to implement, together with ongoing input and maintenance costs (Burgess, Incoll, Hart, & Beaton, 2003). Additional time costs may be associated with maintenance or harvesting (Lasco, Delfino, Catacutan, Simelton, & Wilson, 2014). Optimal implementation may increase primary crop or livestock production, though often yields are reduced owing to light and water competition (Burgess & Rosati, 2018; Lorenz & Lal, 2014). Timber, leaves and fruits may be harvested from trees for use or sale (Eichhorn et al., 2006; Palma et al., 2018).

##### Private non‐financial barriers (risk, behavioural; resilience)

Perceived risk of yield loss or other negative impacts on the production system may represent a behavioural barrier, and the long‐term timescale may also engender reluctance to commit (Mbow et al., 2014). Agroforestry systems typically induce a microclimate effect, improving the climate change adaptability of vulnerable agroecosystems (Lasco et al., 2014; Mbow et al., 2014), as well as improving resilience to pests, diseases, erosion and heat stress (Lasco et al., 2014), though may contribute to increased bushfire incidence or severity (Lorenz & Lal, 2014).

##### Environmental externalities (ecosystem)

Agroforestry should induce ecosystem benefits, including biodiversity, habitat connectivity and water quality (Jose, 2009).

##### Socio‐economic externalities (input demand, output supply)

Establishment and maintenance of agroforestry systems may qualitatively change system input demands, and supply of outputs from the system may change qualitatively as a result of agroforestry by‐products (e.g. fruits, wood; Lasco et al., 2014).

## 4 MODELLING TO OPERATIONALISE SCS

The practices identified and described in this paper are heterogeneous between different regions, climates and production systems in terms of their technical and socio‐economic viability. Facilitation of SCS in agricultural soils is not, therefore, the identification of universally applicable measures, but the development of methodologies which can be used to identify appropriate measures in different environments and production systems. This section discusses how extant methodologies may be applied to identify measures for different production systems, regions and climates.

Assessing a measure's direct impact on the agroecosystem requires the consideration of possible effects on soil biochemistry, plant growth and the loss of C and key nutrients. The range of models suitable for this purpose can be considered to form a continuum of complexity, bounded, on one edge, by simpler models built on empirical relationships and, on the other, by process‐based models seeking to describe the underlying mechanisms in detail. In general, an empirical model connects the system's main drivers (e.g. climate, soil conditions) to its outputs (e.g. soil CO_2_ fluxes) using fewer intermediate nodes (e.g. biochemical subprocesses) than a more process‐based model. This spectrum is not a dichotomy; empirical models are, usually, less data demanding than process models, and due to the fact that our knowledge on certain soil processes remains limited, many process models also depend on empirical submodels to some extent (Brilli et al., 2017; Butterbach‐Bahl, Baggs, Dannenmann, Kiese, & Zechmeister‐Boltenstern, 2013). Here, we review of how the SCS practices, measures and pathways defined in this assessment may be characterised in existing biogeochemical models, considering the range of the described complexity spectrum.

Crop residue retention is one of the most frequently examined SCS measures in relevant model‐based studies (Turmel et al., 2015). Any portion of the crop biomass can be left on the field as residue after harvest, with a fraction of that C eventually entering the soil system. While the complexity of a model's soil C architecture can vary greatly, a typical model includes a number of discrete C pools each with a specific C decomposition potential, from inert to very labile. How residues‐based C is allocated to the different pools varies depending on the model's level of descriptive detail; the most common approaches make use of crop‐specific allocation rules, or discriminate based on residue C:N ratio and lignin content (Liang, Yuan, Yang, & Meng, 2017; Thevenot, Dignac, & Rumpel, 2010). The description of C turnover in each model pool can be controlled by factors such as soil moisture, temperature and the size of the soil's microbial pool (if considered; Smith et al., 2010; Taghizadeh‐Toosi et al., 2014; Wu & Mcgechan, 1998). If the model is able to describe N cycling processes, then each pool's C:N ratio is also used in C turnover‐related process. Finally, a model might also be able to consider the impact of residues cover on soil temperature and moisture under no till conditions.

Tillage regimes are also frequently modelled as SCS measures. Of particular interest, this respect is the way a model describes the discretisation of the soil profile. Simple models may treat the modelled soil as a uniform volume or discretise it into very few layers (e.g. a top and a deeper layer). Detailed and process‐oriented models tend to use more layers (Taghizadeh‐Toosi, Christensen, Glendining, & Olesen, 2016). More detailed models will be able to consider how the vertical movement of C, nutrients and water is modelled. With this structure, the simplest approach in modelling tillage effects is to use a tillage factor and directly adjust how much C is lost after each tillage event (Andales, Batchelor, Anderson, Farnham, & Whigham, 2000; Chatskikh, Hansen, Olesen, & Petersen, 2009). Depending on the model's soil C pool architecture, this factor can be used to adjust either the total soil CO_2_ or its constituents (i.e. decomposition and maintenance CO_2_; Fiedler, Buczko, Jurasinski, & Glatzel, 2015). The more process‐oriented approach is to consider the effect of tillage to the physical (i.e. bulk density) and chemical (i.e. C:N due to residues incorporation) properties of the soil layers that tillage disturbs directly (Leite, De Sá Mendonça, De Almeida MacHado, Fernandes Filho, & Lima Neves, 2004). This readjustment of BD and soil‐pool CN ratios has consequences on all other aspects of the soil's C dynamics (e.g. decomposition, microbial activity, etc).

The modelling of soil erosion has a relatively long history, with more recent links to soil C (Laflen & Flanagan, 2013). While water, tillage and wind are major drivers of soil erosion, most existing erosion models are essentially models of water erosion with tillage and wind effects underexamined (Doetterl et al., 2016). The universal soil loss equation (USLE) and its revised version (RUSLE) are widely used empirical erosion models. These models use empirical factors to consider (a) the soil's rainfall‐induced erodibility; (b) the influence of crop cover and management; and (c) the role of slope (Panagos, Meusburger, Ballabio, Borrelli, & Alewell, 2014). Recent studies have attempted to couple USLE/RUSLE to simpler and more process‐oriented soil‐C models in order to describe erosion‐caused losses of soil C (Wilken, Sommer, Oost, Bens, & Fiener, 2017). Modelling is complicated by (a) the episodic nature of erosion processes (Fiener et al., 2015); (b) feedback loops between SOC, stability of soil aggregates and soil erodibility (Ruis & Blanco‐Canqui, 2017); and (c) small‐scale heterogeneity of erosion processes (Panagos et al., 2016).

In contrast to soil erosion, the modelling of agroforestry systems has a rather limited history. The fundamental modelling approach, especially in studies at larger spatial scales, is to attribute certain fractions of the simulated area to crops or grass and trees and model each ecosystem element independently. This approach does not consider the possible impacts that tree–crop interactions may have (Luedeling et al., 2016), and some process‐oriented models can address this by simulating the impacts of trees on the agroecosystem microclimate (e.g. solar interception, wind speed; Smethurst et al., 2017).

The modelling of nutrient and water management in agroecosystems depends on the ability of a model to consider the role of nutrients and water on soil C decomposition processes (Li et al., 2016; Zhang et al., 2015). As mentioned, soil C modelling is often based on adjusting soil C decomposition rates according to the soil's N content, its temperature and its moisture level. More detailed models can consider the role of soil O_2_ levels, cation exchange capacity and pH and use them, directly or indirectly, to define the amount and type of soil organisms.

Crop rotations modelling is, generally, straightforward. Nevertheless, the robustness of modelling rotations depends on the ability of the model to discriminate between crops in terms of their biomass potential, the partitioning of growing biomass and their nutrient and water demands (Li et al., 2016; Zhang et al., 2015). In this context, it is good knowledge on sowing and harvesting dates, crop varieties and fertilisation‐ and irrigation‐related parameters (e.g. quantities, timings) that will determine how realistically crop rotations and their impacts on soil C are modelled.

The modelling of grasslands and their management has similarities with that of crop rotations in part because of dependence on difficult to obtain input data (e.g. animal type, grass variety or mixture; Li, Liu, Wu, Niu, & Tian, 2015; Sándor et al., 2016). The simplest way to describe the impacts of animal stocks on soil C is based on adjusting the amount of grass (and thus aboveground C and nutrients) that is removed from the ecosystem via grazing depending on animal type and size (Irving, 2015). However, the movement of grazed biomass C and N through the animal and to the soil's surface is itself a complex part of the grazed grassland ecosystem. Livestock presence also affects soil texture and compaction (Li, Snow, & Holzworth, 2011). N fixation by sward legumes is another grass‐based GGR technique, with N fixation modelling based on the assumptions that (a) fixation is activated if plant N demand is not met; (b) N fixation capabilities are related to the growing grass variety; and (c) that the amount of N fixed is proportional to the size of the plant's root system (Chen et al., 2016; Gopalakrishnan, Cristina Negri, & Salas, 2012).

Whether fires are natural or man‐made, spatial context is key for fire modelling. Empirical models utilise a simplistic concept of ‘fire probability’; a function of available combustible plant material, fire season length, soil moisture and fuel extinction moisture content (Hantson et al., 2016). Process‐based models are also based on this concept but may parameterise the spread and intensity of fire in more detail (Thonicke et al., 2010). The description of the impacts of fire on vegetation varies between models, but it is typically estimated on the basis of fuel availability (i.e. plant biomass), plant specific mortality and regeneration. In this context, the modelling approach is, in essence, empirical but process models can go into some detail by considering the role of bark thickness, tree diameter and resprouting (Kelley, Harrison, & Prentice, 2014).

While biochar application is a promising SCS measure, lack of experimental data means few models can simulate it effectively (Sohi, 2012; Tan, Lin, Ji, & Rainey, 2017). The empirical modelling approach treats biochar as a quantity of C made up by different fractions, each with a specific degree of decomposability. The biggest part of biochar C is considered as being protected against further decomposition while the rest can be more or less exposed to decomposition (Woolf, Amonette, Street‐Perrot, Lehmann, & Joseph, 2010). The more process‐based description is based on the same principles but considers the impacts of biochar to the soil's physical (i.e. bulk density, water retention) and chemical (i.e. CEC, N retention) properties (Archontoulis et al., 2016). These physicochemical properties are, in turn, influencing the turnover of the soil's different C pools.

For all measures, their implementation in global agroecosystems is likely to modify both land management practices and system outputs. Life cycle assessment (LCA) is a standardised methodology (ISO 14044‐2006; ISO 14040‐2006) for estimation of environmental consequences resulting from system modification (CML, 2015; Goedkoop et al., 2009; Goglio, Smith, Worth, et al., 2018). However, there is no standardised procedure for the assessment of SCS in LCA; apart from coupling with the biophysical approaches described, LCA analyses may also consider the consequences of SCS on local, regional and global markets; given the holistic nature of many SCS practices, implementation may cause variation in system outputs (Dalgaard et al., 2008; Schmidt, 2008). A consequential LCA achieves this by considering the marginal actors affected by a market change (Ekvall & Weidema, 2004; Schmidt, 2008) and the potential consequences of a particular production system influencing the world market (Anex & Lifset, 2014; Plevin, Delucchi, & Creutzig, 2014). This complex approach requires the identification of marginal data (e.g. competitive energy and material suppliers), whose availability determines the level of uncertainty of the assessment (Ekvall & Weidema, 2004).

The main elements of the biophysical modelling processes reviewed here, as they relate to the specific measures defined in this assessment, are summarised in Table 2. Table 2 also summarises the key impacts of each measure likely to be influential in LCA assessments of their implementation in global agroecosystems.

**Table 2:** Summary of key biophysical modelling elements and LCA considerations for the defined SCS measures assessed. These elements are generalisations based on the literature review in Sections 3 and 4

Practice	Measure	Key elements for biophysical agroecosystem models	Key elements for LCA^a^
Soil structure management	Prevent or control soil erosion	Fate of eroded soil C Impact of erosion on primary productivity Impact of control measures on erosion	Agricultural production impacts Environmental impact(s) of physical erosion control structures and/or erosion control practices
	Optimise fire frequency and timing	Impact of fire on agroecosystem productivity Impact of fire on mineralisation of soil C stocks	Agricultural production impacts CO_2_ released from burn Non‐CO_2_ climate forcers released from burn
	Practise reduced or zero tillage	Impact of soil structure/aggregation on mineralisation of soil C stocks Impact of tillage regime on primary productivity	Agricultural production impacts Change in energy usage for tillage practice Environmental impact(s) of required capital items
Grazing land management	Optimise stocking density	Impact of grazing density on agroecosystem biomass retention Physical impact of livestock on soil structure Impact of soil structure on microbial mineralisation	Agricultural production impacts Impact of stocking density on livestock direct emissions
	Renovate unimproved pasture	Impact of new sward on agroecosystem primary productivity and N fixation Impact of renovation on soil C stocks	Agricultural production impacts Impact of sward change on livestock direct emissions Environmental impact(s) of sward renovation inputs and agrochemicals
Improved rotation management	Extend perennial phase of crop rotations	Impact of perennial rotation phase on soil C inputs, losses and N fixation Impact of annual phase on soil C inputs, losses and N fixation	Agricultural production impacts Change in input/agrochemical usage for new rotation Change in energy requirements for cultivation
	Implement cover cropping	Impact of cover crop on soil C inputs Impact of cover crop on mineralisation of soil C stocks	Agricultural production impacts Environmental impact(s) of energy, input and agrochemical usage changes resulting from cover crop
Inorganic resource management	Optimise soil synthetic nutrient input	Impact of nutrient availability on crop primary productivity Impact of increased primary productivity/nutrients on mineralisation of C stocks	Agricultural production impacts Energy usage for application Environmental impact(s) of synthetic production, processing and transport
	Practise mineral carbonation of soil	Reaction rate of applied calcium source Agroecosystem primary productivity impact of application	Agricultural production impacts Energy usage from application Environmental impact(s) of product extraction, processing and transport
	Manage soil pH	Impact of application on primary productivity Impact of application on soil structure/aggregation Impact of application on microbial activity/mineralisation of C stocks	Agricultural production impacts Energy usage from application Environmental impact(s) of product extraction, processing and transport
Organic resource management	Optimise use of organic amendments	Impact of application on primary productivity Impact of application on soil structure/aggregation Impact of application on microbial mineralisation of C stocks Net difference between use in system versus other possible uses	Agricultural production impacts Environmental impact(s) of change in fate of organic material Environmental impact(s) of transport Energy usage for application
	Retain crop residues	Impact of retention on primary productivity Impact of retention on microbial mineralisation of C stocks Net difference between use in system versus other possible uses	Agricultural production impacts Environmental impact(s) of change in fate of organic material Energy use for incorporation
	Apply biochar	Net C transfer in biochar production Decomposition rate of biochar Impact of biochar on microbial mineralisation of existing stocks Impact of biochar on primary productivity	Agricultural production impacts Energy usage/production and environmental impact(s) from biochar production, transport and application Environmental impact(s) of change in fate of organic material
Soil water management	Optimise irrigation	Impact of soil water content on primary productivity Impact of soil water content on microbial mineralisation of C stocks	Agricultural production impacts Environmental impact(s) of required capital items Direct water usage and environmental impact(s) of abstraction
Woody biomass integration	Implement agroforestry systems	Impact of woody biomass on belowground C Sequestration of C in woody biomass Impact of tree–understorey interactions on understorey productivity	Agricultural production impacts, including tree‐based by‐products Environmental/energy use impacts of agroforestry system implementation, maintenance and harvesting

a In addition to direct, land‐based GHG fluxes (CO_2_, N_2_O, CH_4_) presumed quantified by biophysical agroecosystem models.

## 5 POLICY RELEVANCE AND CONCLUSIONS

The potential of SCS in offsetting emissions and supporting food security is now recognised in global policy initiatives such as the 4 per mille international research program (Minasny et al., 2017). This assessment has identified a range of SCS practices which can be considered to be an effective route to GGR in global agricultural soils, and to critically assess the biophysical, economic and social impacts of these measures and their implementation in global systems. While not unique in this respect (e.g. Chenu et al., 2019), in providing a framework for the application of existing knowledge and methodologies to the challenge of local‐ and regional‐scale SCS implementation, this assessment represents a novel approach in facilitating SCS. Recognition, incentives or credits for these practices require robust monitoring, reporting and verification procedures, and defining a standardised framework for the assessment of these measures is a useful step towards implementation of such a system.

Calls for the agricultural economy to reflect ecosystem services provided by soil are numerous (e.g. Lal, 2016; Panagos et al., 2016; Thamo & Pannell, 2016), and in practice amount to rewarding farmers for implementation of SCS practices, whether through direct subsidy (i.e. payments for public goods) or through the development of private offset markets (Kroeger & Casey, 2007). The former is already happening and includes the Australian Government's Carbon Farming Initiative (Bispo et al., 2017). In the European Union, there are ongoing discussions about how SCS can be included in payments related to the Common Agricultural Policy, though problems in terms of monitoring compliance and evaluation must be addressed. The same problems hinder the development of carbon credit markets or other potential payment methods, which are currently more piecemeal, and require an understanding of the technical, economic and social viability of SCS practices. In following the approach taken in this assessment, we have defined a framework which can be used to structure extant knowledge and approaches in fulfilling these requirements. Particularly, a distinction emerged in the process of this assessment between (a) measures which represent the implementation of a management action specifically for the purpose of inducing SCS in the agroecosystem; and (b) those which represent the optimisation of elements of the agricultural system which are either common practice (e.g. synthetic or organic nutrient regimes) or an inherent part of the agroecosystem (e.g. stocking density). Those in the latter group are less well represented in the literature by comparison, and are challenging to discuss, in that they can be defined only against the system in which they are to be implemented, and hence require detailed understanding of the management practices and biophysical processes in that system. The modelling approaches reviewed (Section 4), coupled with good quality local or regional baseline data, will be necessary to actually define these measures in such a way that they may be implemented in agricultural systems.

Another important distinction which emerges exists between measures which primarily facilitate C storage, as opposed to those which directly induce sequestration (defined as in Chenu et al., 2019). Measures falling under Section 3.5 can be categorised in the former way, and are highly dependent on assumptions made about the alternative fate of the source material, and its comparative residence time in the soil C pool. The availability of this material also places limits on the maximum SCS which can be achieved via this measure as well as challenges relating to supply and demand (e.g. Schlesinger & Amundson, 2019). All these measures induce externalities relating to inputs and outputs from the agricultural system, the market effect of which is challenging to predict (Plevin et al., 2014).

Optimism relating to SCS for GGR is high (Minasny et al., 2017) and the surrounding literature is developing at a fast pace (Minx et al., 2017). In identifying a gap between global‐scale assessments (e.g. Smith, 2016) and measure‐based or region‐specific analyses, this paper brings together a novel combination of discrete SCS measures with a thorough, literature‐based framework for the alignment of extant knowledge and methods, and the objective and quantitative assessment of SCS in global agricultural systems. This is a crucial step in translating existing science into policy able to incentivise farmers to implement SCS measures (Bispo et al., 2017; Lal, 2016; Smith, 2016).
